# Insights into the adaptive response of the plant-pathogenic oomycete *Phytophthora capsici* to the fungicide flumorph

**DOI:** 10.1038/srep24103

**Published:** 2016-04-06

**Authors:** Zhili Pang, Lei Chen, Wenjun Mu, Li Liu, Xili Liu

**Affiliations:** 1 Department of Plant Pathology, College of Agriculture and Biotechnology, China Agricultural University, Beijing, P. R. China; 2 College of Forestry, Beijing Forestry University, Beijing, P. R. China; 3 Zhengzhou Tobacco Research Institute of CNTC, P. R. China

## Abstract

*Phytophthora capsici* is an important oomycete plant pathogen that causes significant losses worldwide. The carboxylic acid amide fungicide flumorph has shown excellent activity against oomycete plant pathogens. Despite its potential, there remains concern that the sexual reproduction of oomycete pathogens, which results in genetic recombination, could result in the rapid development of resistance to flumorph. The current study utilized an iTRAQ (isobaric tags for relative and absolute quantitation) based method to compare differences between the proteome of the parental *P. capsici* isolate PCAS1 and its sexual progeny S_2_-838, which exhibits significant resistance to flumorph. A total of 2396 individual proteins were identified, of these, 181 were considered to be associated with the adaptive response of *P. capsici* to flumorph. The subsequent bioinformatic analysis revealed that the adaptive response of *P. capsici* to flumorph was complex and regulated by multiple mechanisms, including utilising carbohydrate from the host environment to compensate for the cell wall stress induced by flumorph, a shift in energy generation, decreased amino acids biosynthesis, and elevated levels of proteins associated with the pathogen’s response to stimulus and transmembrane transport. Moreover, the results of the study provided crucial data that could provide the basis for early monitoring of flumorph resistance in field populations of *P. capsici*.

*Phytophthora capsici* was first reported by Leon H. Leonian in 1922^1^, and is currently regarded as one of the 10 most important oomycete pathogens in molecular plant pathology^2^. This devastating pathogen has a global distribution and can infect more than 45 species of plants including both crops and weed species^3^,^4^,^5^,^6^, causing crown, root, and fruit rot^7^,^8^,^9^, which lead to significant economic losses every year^2^,^5^,^10^. Although, crop rotation and other management tools contribute to the control of diseases caused by *P. capsici*, in practice there is a heavy reliance on fungicides^11^,^12^. The carboxylic acid amide (CAA) fungicide flumorph, 4-[3-(3,4-dimethoxyphenyl)-3-(4-fluorophenyl)-1-oxo-2-propenyl] morpholine, which was developed by the Shenyang Research Institute of Chemical Industry of China in 1994^13^, has been patented in China (ZL.96115551.5), the United States (US6020332), and Europe (0860438B1). It is currently registered for the control *P. capsici*, *Phytophthora infestans*, *Pseudoperonospora cubensis*, and *Plasmopara viticola* in China, and remains an effective fungicide to control diseases caused by *P. capsici*^14^.

The sexual reproduction of *P. capsici*^15^ plays an important role in its disease cycle initiating infection in host plants^5^,^16^, while the resulting genetic recombination can contribute to the development of isolates that exhibit complete insensitivity to certain fungicides^17^. *P. capsici* is a heterothallic pathogen that produces two mating types, A1 and A2, and it has been shown that the co-occurrence of both mating types in regions of the United States, South Africa, and the northern provinces of China, can facilitate frequent outcrossing and increase the risk of resistance developing^18^,^19^,^20^,^21^. Previous studies have shown that flumorph resistance in *P. capsici* is controlled by two dominant genes, which implies that once resistance has developed it could rapidly spread through a population via both sexual and asexual reproduction^22^.

Proteomics has become a useful tool for studying the biological effects of fungicides. For example, 2-DE has been used to investigate the global response of *Saccharomyces cerevisiae* in the early stages of exposure to mancozeb^23^, while MALDI-TOF-MS/MS has been used to study the mode of action of the fungicide JS399-19 in *Fusarium graminearum*^24^, and iTRAQ (isobaric tags for relative and absolute quantitation) technology to study the effect of pyrimorph in *P. capsici*^25^. The current study adopted a similar approach, using iTRAQ to compare the response of a wild-type parental *P. capsici* isolate (PCAS1) and its flumorph resistant sexual progeny (S_2_-838). The proteomics data produced would hopefully provide a greater understanding of the adaptive mechanisms associated with flumorph resistance in *P. capsici*, as well as highlighting target proteins for not only the early monitoring of flumorph resistance, especially that associated with sexual reproduction, but also for the design of novel fungicides.

## Results

### Overview of quantitative proteomics analysis

A total of 2396 individual proteins with at least one unique peptide and protein scores >20 were identified from the wild-type (PCAS1) and flumorph-resistant (S_2_-838) isolates of *P. capsici* cultured in the presence or absence of flumorph (1.5 μg/ml or 100 μg/ml, respectively) using iTRAQ-LC-MS/MS analysis (identified protein and peptide information, Supplementary Table S1, S2).

### Effect of flumorph on protein levels

In total, 189 and 26 proteins were found to be significantly altered in PCAS1 and S_2_-838, respectively (Supplementary Table S3, S4). Of the 189 proteins detected in the wild-type isolate PCAS1, a total of 80 were up-regulated, and the other 109 down-regulated. In contrast, the flumorph-resistant isolate S_2_-838 was much less affected with only 21 up-regulated proteins and 5 down-regulated ones.

### Identification of candidate proteins for the adaptive response of *P. capsici* to flumorph

**Figure 1:**
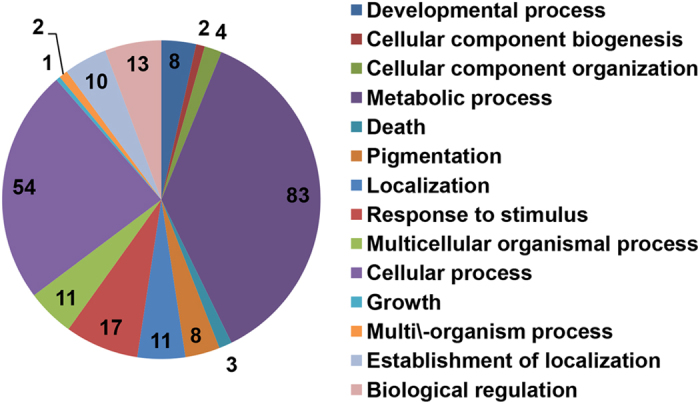
GO annotation of candidate proteins associated with the adaptive response of *P. capsici* to flumorph. Numbers indicate the number of proteins categorized into each functional group.

**Figure 2:**
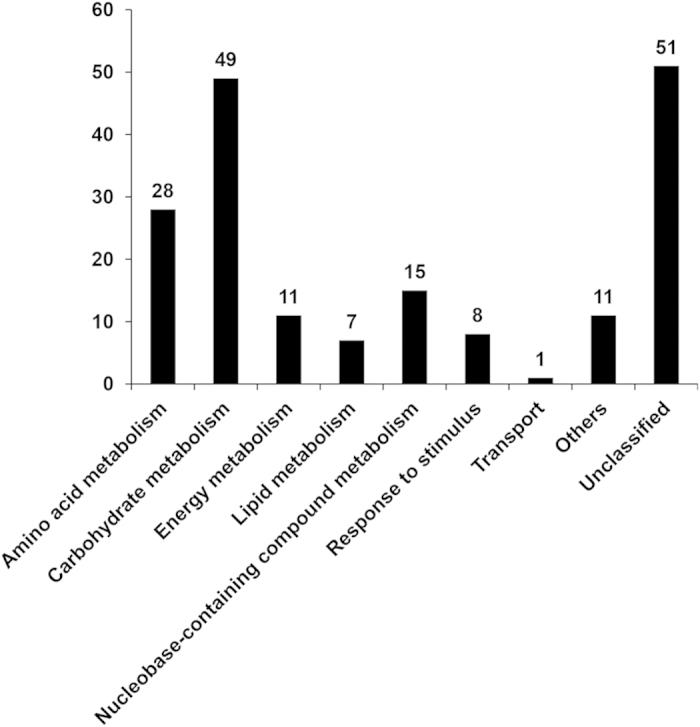
Distribution of candidate proteins associated with the adaptive response of *P. capsici* to flumorph as categorized by KEGG pathway analysis. Numbers indicate the number of proteins in each category.

It was found that 181 proteins were associated with the adaptive response of *P. capsici* to flumorph, with altered levels of abundance in the wild-type isolate PCAS1, but not in the flumorph-resistant isolate S_2_-838, when comparing the control cultures to those treated with flumorph (Table 1). The subsequent GO analysis categorized these proteins into 14 functional groups according to their biological activity (Fig. 1). The majority of the proteins fell into just two categories metabolic process (83) and cellular process (54). The other proteins fell into 12 categories including developmental process, cellular component biogenesis, cellular component organization, death, pigmentation, localization, response to stimulus, multicellular organismal process, growth, multi/-organism process, establishment of localization, and biological regulation. However, it should be noted that a single protein can be assigned to more than one category. Metabolic pathway enrichment analysis was then performed by matching the proteins with altered abundance to annotated proteins in the KEGG Pathway database. Although it was not possible to classify a large number of the proteins (51), the majority were assigned to a diverse range of metabolic pathways, including amino acid metabolism, carbohydrate metabolism, energy metabolism, lipid metabolism, nucleobase-containing compound metabolism, response to stimulus, transport, and other metabolic pathway (Fig. 2).

## Discussion

An iTRAQ-LC-MS/MS approach was used to investigate the effect of the CAA fungicide flumorph on wild-type and resistant isolates of *P. capsici*. Altogether, 2406 individual proteins were identified, a number of 189 and 26 proteins were found to have altered levels of abundance in response to flumorph stress in PCAS1 and S_2_-838, respectively. One reason for the big difference in the number of differentially expressed proteins between the isolates PCAS1 and S_2_-838 can be contributed to the different genetic background. Compared to the wild-type isolate, the point mutations in cellulose synthase 3 caused the resistance to CAA fungicides in the mutant^25^,^26^. The 181 proteins related with genetic background of flumorph resistance were identified as candidates for the adaptive response of *P. capsici* to flumorph. The subsequent GO analysis categorized the proteins into 14 biological processes. However, KEGG pathway analysis indicated that 51 of the proteins have yet to be assigned metabolic pathways and therefore provide little insight into the effect of flumorph, although they could be utilized as candidate proteins for future study. The roles of the remaining 130 proteins were discussed below.

Carbohydrate metabolism was the pathway most affected by flumorph and was associated with the altered abundance of 46 proteins, of which 10 and 36 were up-regulated and down-regulated in response to flumorph, respectively. Two of the up-regulated proteins, Glucan-1,3-beta-glucosidase (Accession number: 262097763, and 262098611) and exo-1,3-beta-glucanase are involved in the break down glucan to release glucose^27^,^28^. Previous investigations into the mode of action of CAA fungicides have revealed that mandipropamid and pyrimorph can inhibit cell wall biosynthesis in *P. infestans* and *P. capsici*, respectively^25^,^26^. Given that the cell walls of oomycetes mainly consist of cellulose and 1,3-β-glucans^29^, the increased abundance of glucan-1,3-beta-glucosidase and exo-1,3-beta-glucanase suggested that *P. capsici* utilized carbohydrate from the host environment to compensate for the cell wall stress induced by flumorph. Similarly, the increased abundance of PHYSODRAFT_261542, PHYSODRAFT_302104, and PHYSODRAFT_565653, which are involved in glucan biosynthesis^30^, could also represent an adaptation to the cell wall stress induced by flumorph. The most down-regulated proteins were associated with glycolysis and the citric acid (TCA) cycle, which are involved in the utilization of glucose and other carbohydrates to generate ATP. However, interestingly, all the proteins involved in lipid metabolism, which can also result in the production of large amounts of energy, were up-regulated in response to flumorph. The altered level of these flumorph-responsive proteins suggested that flumorph might induce a redistribution of the metabolic processes associated with energy production. This hypothesized shift of energy generation from glycolysis and the citrate cycle to lipid metabolism could allow for the redistribution of glucose or carbohydrate in response to the inhibition of cell wall biosynthesis caused by flumorph.

Although the majority of the proteins with altered levels of abundance were associated with energy metabolism, a significant number of proteins (9 up-regulated, 20 down-regulated) were associated with amino acid metabolism. Several of the down-regulated proteins were found to play a role in the biosynthesis of amino acids and proteins including argininosuccinate lyase^31^ and the hypothetical protein PHYSODRAFT_485399^30^, which are involved in arginine biosynthesis; hypothetical protein PHYSODRAFT_471713^30^ and 5-methlytetrahydropteroyltriglutamate-homocysteine methyltransferease^32^, which play a role in methionine biosynthesis; and cysteine synthase, which participates in cysteine biosynthesis^33^; as well as the eukaryotic translation initiation factor 3^34^, all of which might play important roles in protein synthesis. It is therefore possible that a reduced rate of global protein synthesis could be another adaptive response of *P. capsici* to flumorph stress as the pathogen attempts to maintain the fidelity of its protein biosynthesis. Similar results have also been observed in the response of *P. capsici* to another CAA fungicide, pyrimorph^25^.

It was also interesting that some proteins with altered levels of abundance were associated with response to stimulus and signal transduction. For example, alkaline phosphatase^35^ was up-regulated in response to flumorph. This important hydrolase enzyme catalyzes dephosphorylation during the post-translational modification of proteins^36^. Dephosphorylation and phosphorylation of S, T, Y and H residues are the best characterized modifications associated with the reversible, activation and inactivation of enzyme activity and the modulation of molecular interactions in signaling pathways^37^. It was also found that elicitin protein was up-regulated. Elicitin superfamily of proteins are structurally related to extracellular proteins that induce hypersensitive cell death and other biochemical changes associated with the defense response^38^,^39^,^40^. The up-regulation of alkaline phosphatase and elicitin in *P. capsici* therefore suggested that signal transduction was an important factor in responding to flumorph stress.

It was also found that a protein ABCA1 (from lipid exporter family) associated with transmembrane transport, an ATP binding cassette A (ABCA) superfamily protein was up-regulated in *P. capsici* in response to flumorph. Members of the ABCA family proteins have also been implicated in the adaptation to environmental changes in the free-living state of *Phytophthora sojae*^41^. The activation of this transporter could confer significant selective advantage to *P. capsici* isolates responding to flumorph stress in their environment.

Taken together, these results indicated that the adaptive response of *P. capsici* to flumorph was complex and regulated by multiple pathways, including utilising carbohydrate from the host environment to compensate for the cell wall stress induced by flumorph, a shift in energy generation from glycolysis and citrate cycle to lipid metabolism, decreased amino acids biosynthesis, and elevated levels of proteins associated with the pathogen’s response to stimulus and transmembrane transport. The proteomic data produced in the current study could provide important insight into the adaptive response of *P. capsici* to flumorph that would be useful for monitoring the emergence of resistance in field populations.

## Material and Methods

### Strains, medium, and growth conditions

The wild-type *P. capsici* isolate PCAS1 (P1314, mating type A1), which was originally collected from diseased green pepper (*Capsicum annuum* L.), was kindly provided by Professor Michael Coffey (University of California, Riverside, USA), while the sexual progeny S_2_-838 was generated in a previous study^22^. The EC_50_ values (the effective concentration for 50% inhibition of mycelial growth) for flumorph in the two isolates was approximately 1.5 μg/ml and 100 μg/ml (the maximal soluble concentration of flumorph), respectively (Supplementary Fig. S1). Potato dextrose agar (PDA) or potato dextrose broth (PDB) was used for routine maintenance of the cultures, which were dark incubated at 25 °C.

### Sample preparation for proteomic analysis

Mycelium collected from 4-day cultures growing on PDA medium with a cellophane sheet were harvested and used to inoculate PDB medium in the presence or absence of flumorph (1.5 μg/ml or 100 μg/ml for the wild-type and resistant isolates, respectively). Each treatment has 10 biological replicates. After 24 hours dark-incubation at 25 °C with shaking, the mycelia were collected by filtration, washed profusely with sterile distilled water, dried, and ground thoroughly in liquid nitrogen. The resulting samples were stored at −80 °C until required. The experiment was repeated three times. The protein was extracted from approximately 100 μg of each sample, which were resuspended in 1 ml lysis buffer [8 M urea, 30 mM HEPES, 5 mM TCEP, and 2 mM EDTA] with the aid of a sonicator (Branson^®^ Sonifier 250, BRANSON Ultrasonics Corporation, Danbury, U. S. A.). Any undisrupted cells were removed by centrifugation with the supernatant being transferred to fresh tubes. The samples were then incubated at 60 °C for one hour before the addition of 1% iodoethanol for another one hour in dark. The proteins were precipitated overnight in a freezer using 4 volumes of cold acetone, before being collected by centrifugation at 20000 rpm for 20 min. Finally, the proteins were dissolved in 50 mM triethylammonium bicarbonate (TEAB) containing 1% SDC.

### Isobaric Tags for Relative and Absolute Quantitation (iTRAQ) and Liquid Chromatography Coupled with Tandem Mass Spectrometry (LC−MS/MS) Analysis

Since technical variation of iTRAQ measurements was demonstrated to be on the order of 20%^42^, pooling samples were used to produce such biases^43^,^44^. In our study, ten biological replicates for each sample were pooled together to produce one sample, and 100 μg aliquots digested with 1 μg/μl trypsin overnight at 37 °C. After being lyophilized the samples belonging to individual treatments were labeled with different iTRAQ reagents (Applied Biosystems, Foster City, CA.) according to the protocol of the manufacturer. The untreated PCAS1 control was labeled with 114, while the PCAS1 treated with flumorph, S_2_-838 control and S_2_-838 treated with flumorph were labeled with 115, 116 and 117, respectively. The labeled peptides were then combined and dried in a vacuum concentrator. The first dimension of the 2D-LC consisted of extensive fractionation of the peptide mixtures by strong cation exchange (SCX) chromatography to improve proteome coverage. Briefly, the dried samples were reconstituted in buffer A [25% (v/v) acetonitrile (ACN), 10 mM potassium phosphate; pH adjusted to 3.0] and loaded onto a Lumn A column (4.6-mm i.d. × 100-mm length, 5 μm, 100 Å; Phenomenex, USA). The column was equilibrated for 10 min in buffer A before the peptides were eluted at a flow rate of 1 mL/min using buffer B [25% (v/v) ACN, 2 M potassium chloride, 10 mM potassium phosphate; pH adjusted to 3] at a succession of increasing gradients 0–30% for 15 min, followed by 30–100% for 15 min, and finally 100% buffer B for 10 min. A total of fifteen peptide fractions were collected, which were then dried using a SpeedVac centrifugal vacuum concentrator and purified on a strata-X C18 column (Phenomenex, USA) prior to mass spectrometry (MS) analysis.

The LC-MS/MS experiments were performed using an integrated system consisting of a Q Exactive™ Mass Spectrometer (Thermo Fisher Scientific, USA) coupled with a nanoflow HPLC system (Easy nLC, Proxeon Biosystems, now Thermo Fisher Scientific, USA). Each fraction was reconstituted in 0.1% formic acid before being injected into the LC-MS/MS system. The samples eluted from the trap column were separated on a PepMap C18 column (100 mm × 75 mm, 300-Å pore size, 5 μm particle size, Thermo scientific, USA) at a rate of 400 nL/min, using 0.1% formic acid as solvent A and 0.1% formic acid in acetonitrile as solvent B, in increasing gradients: 0.1–5% B (0–10 min), 5–30% B (10–40 min), 30–60% B (40–45 min), 60–80% B (45–48 min), 80% B (48–55 min), 80–0.1% B (55–65 min). The eluting peptides were sprayed into the mass spectrometer at an ion spray voltage of 1800 eV, and their MS/MS spectra acquired using automated data-directed switching between the MS and MS/MS modes. The five most abundant signals from each survey scan (350–2000 m/z range) were selected by charge state, and the collision energy applied accordingly for the sequential MS/MS fragmentation scanning as described previously^45^. The entire experiment was conducted three times.

### Data Processing and Analysis

The raw MS/MS data were merged and transformed using the Proteome Discoverer software package (version 1.3; Thermo Fisher Scientific, USA)^46^ before Mascot version 2.3.01 (Matrix Sciences, Ltd., London, UK) was used to identify and quantify the individual proteins according to sequences contained in the NCBI Oomycetes database using the following settings: trypsin specific digestion with one missed cleavage allowed, peptide tolerance of 15 ppm, MS/MS tolerance of 20 mmu, iTRAQ 4-plex for peptide N-t and Lys as fixed modifications, and in variable mode, iTRAQ 4-plex on Tyr, oxidized Met and methylthio on Cys. The false positive rate, which was checked using a concatenated target-decoy database search strategy, was set to be less than 1%. Only proteins with at least one unique peptide and having protein scores of more than 20 were initially recorded. Only proteins with two or more peptides were used for the quantitative analysis. The LIBRA tool from the TPP software^47^ was used for protein quantification using the default parameters. The relative abundance of proteins in the different treatments were calculated from three replicates using the log2 of the iTRAQ ratios, which were normalized before the standard deviations from the corresponding normal distributions of ratios were used to determine the cutoff point of the experiment^48^. Proteins whose average ratios fell outside a standard deviation of ±1 from the global mean were considered to have differential abundance. Gene Ontology (GO) annotation was conducted using information retrieved from the UniProt and BGI WEGO (http://wego.genomics.org.cn) databases^49^, while the pathway enrichment analysis was conducted using the Kyoto Encyclopedia of Genes and Genomes (KEGG) Pathway database^50^.

## Additional Information

**How to cite this article**: Pang, Z. *et al.* Insights into the adaptive response of the plant-pathogenic oomycete *Phytophthora capsici* to the fungicide flumorph. *Sci. Rep.* **6**, 24103; doi: 10.1038/srep24103 (2016).

## Supplementary Material

Supplementary Information

Supplementary Table S1

Supplementary Table S2

Supplementary Table S3

Supplementary Table S4

## Figures and Tables

**Table 1:** Candidate proteins identified by iTRAQ analysis for the adaptive response of *P. capsici* to flumorph.

Accession	Description	Pathway
348690896	hypothetical protein PHYSODRAFT_553624	Amino acid metabolism
348690401	Hypothetical protein PHYSODRAFT_553293	Amino acid metabolism
348689700	Hypothetical protein PHYSODRAFT_294643	Amino acid metabolism
348687321	Hypothetical protein PHYSODRAFT_471713	Amino acid metabolism
348685637	Hypothetical protein PHYSODRAFT_326460	Amino acid metabolism
348684607	Hypothetical protein PHYSODRAFT_344705	Amino acid metabolism
348684064	Hypothetical protein PHYSODRAFT_485399	Amino acid metabolism
348683248	hypothetical protein PHYSODRAFT_349622	Amino acid metabolism
348683007	hypothetical protein PHYSODRAFT_284659	Amino acid metabolism
348675955	hypothetical protein PHYSODRAFT_354820	Amino acid metabolism
348675840	Hypothetical protein PHYSODRAFT_260724	Amino acid metabolism
348671280	hypothetical protein PHYSODRAFT_520447	Amino acid metabolism
348668898	Hypothetical protein PHYSODRAFT_564655	Amino acid metabolism
348666294	Hypothetical protein PHYSODRAFT_289076	Amino acid metabolism
262111232	Non-selective Cation Channel-2 (NSCC2) Family	Amino acid metabolism
262110913	Glycine amidinotransferase	Amino acid metabolism
262109966	Protein transporter Sec61 subunit alpha	Amino acid metabolism
262108863	argininosuccinate lyase	Amino acid metabolism
262104624	Glutamyl-tRNA synthetase	Amino acid metabolism
262102276	Cysteine synthase	Amino acid metabolism
262102113	Conserved hypothetical protein	Amino acid metabolism
262102109	Eukaryotic translation initiation factor 3	Amino acid metabolism
262101719	Threonyl-tRNA synthetase	Amino acid metabolism
262100869	Glu/Leu/Phe/Val dehydrogenase family	Amino acid metabolism
262100443	glutathione S-transferase, putative	Amino acid metabolism
262100355	5-methlytetrahydropteroyltriglutamate-homocysteine methyltransferease	Amino acid metabolism
262097641	Conserved hypothetical protein	Amino acid metabolism
262097548	60S ribosomal protein L19-1	Amino acid metabolism
348690023	Hypothetical protein PHYSODRAFT_284519	Carbohydrate metabolism
348688731	Hypothetical protein PHYSODRAFT_284295	Carbohydrate metabolism
348688629	hypothetical protein PHYSODRAFT_343844	Carbohydrate metabolism
348687786	Hypothetical protein PHYSODRAFT_261542	Carbohydrate metabolism
348687768	Hypothetical protein PHYSODRAFT_293395	Carbohydrate metabolism
348687704	hypothetical protein PHYSODRAFT_554034	Carbohydrate metabolism
348686055	Hypothetical protein PHYSODRAFT_354100	Carbohydrate metabolism
348684537	Hypothetical protein PHYSODRAFT_344687	Carbohydrate metabolism
348683824	Hypothetical protein PHYSODRAFT_482943	Carbohydrate metabolism
348683217	Hypothetical protein PHYSODRAFT_358973	Carbohydrate metabolism
348681440	Hypothetical protein PHYSODRAFT_285579	Carbohydrate metabolism
348679829	putative lectin [Phytophthora sojae]	Carbohydrate metabolism
348677650	Hypothetical protein PHYSODRAFT_264166	Carbohydrate metabolism
348676929	Hypothetical protein PHYSODRAFT_559636	Carbohydrate metabolism
348675829	Hypothetical protein PHYSODRAFT_302104	Carbohydrate metabolism
348675658	Hypothetical protein PHYSODRAFT_286325	Carbohydrate metabolism
348674156	Putative exo-1,3-beta-glucanase	Carbohydrate metabolism
348672383	Hypothetical protein PHYSODRAFT_286936	Carbohydrate metabolism
348670337	Hypothetical protein PHYSODRAFT_564447	Carbohydrate metabolism
348670028	Putative carboxylase	Carbohydrate metabolism
348669512	Phosphoglycerate kinase	Carbohydrate metabolism
348667991	Hypothetical protein PHYSODRAFT_526336	Carbohydrate metabolism
348667785	Hypothetical protein PHYSODRAFT_565503	Carbohydrate metabolism
348667135	Hypothetical protein PHYSODRAFT_530509	Carbohydrate metabolism
348666811	Hypothetical protein PHYSODRAFT_565653	Carbohydrate metabolism
348666456	Hypothetical protein PHYSODRAFT_341553	Carbohydrate metabolism
348664576	Hypothetical protein PHYSODRAFT_343277	Carbohydrate metabolism
332985070	Enolase	Carbohydrate metabolism
262112524	Glucokinase, putative	Carbohydrate metabolism
262111992	Fumarate hydratase	Carbohydrate metabolism
262111868	Pyruvate carboxylase	Carbohydrate metabolism
262111867	Pyruvate carboxylase	Carbohydrate metabolism
262111277	D-lactate dehydrogenase	Carbohydrate metabolism
262109936	Dolichyl-diphosphooligosaccharide-protein glycosyltransferase subunit	Carbohydrate metabolism
262109887	Succinate dehydrogenase flavoprotein subunit	Carbohydrate metabolism
262109798	Acetate kinase	Carbohydrate metabolism
262108121	Pyruvate, phosphate dikinase	Carbohydrate metabolism
262107807	Lectin, putative	Carbohydrate metabolism
262104765	Phosphate acetyltransferase	Carbohydrate metabolism
262103650	Fructose 1,6 bisphosphatase	Carbohydrate metabolism
262103560	glyceraldehyde-3-phosphate dehydrogenase	Carbohydrate metabolism
262102812	Malate dehydrogenase	Carbohydrate metabolism
262101165	lectin, putative [Phytophthora infestans T30-4]	Carbohydrate metabolism
262099080	Aldehyde dehydrogenase	Carbohydrate metabolism
262098611	Glucan 1,3-beta-glucosidase	Carbohydrate metabolism
262098605	Phosphoenolpyruvate carboxykinase	Carbohydrate metabolism
262097763	Glucan 1,3-beta-glucosidase	Carbohydrate metabolism
262097378	Phosphoglycerate kinase	Carbohydrate metabolism
262097374	Pyruvate kinase	Carbohydrate metabolism
348689136	Hypothetical protein PHYSODRAFT_552537	Energy metabolism
348683826	Pyrophosphatase	Energy metabolism
348679595	Proton pump, proton transport	Energy metabolism
348675725	Hypothetical protein PHYSODRAFT_346620	Energy metabolism
348673684	Hypothetical protein PHYSODRAFT_562177	Energy metabolism
348671348	Hypothetical protein PHYSODRAFT_287246	Energy metabolism
262109621	Sulfite reductase [NADPH] subunit beta	Energy metabolism
262100029	Plasma membrane H + -ATPase	Energy metabolism
262098159	12-oxophytodienoate reductase, putative	Energy metabolism
262095198	S-formylglutathione hydrolase	Energy metabolism
254576457	NADH dehydrogenase subunit I	Energy metabolism
348690480	Hypothetical protein PHYSODRAFT_553352	Lipid metabolism
348679431	Hypothetical protein PHYSODRAFT_557078	Lipid metabolism
348678066	Putative glycosyl hydrolase family 30 protein	Lipid metabolism
348677854	Hypothetical protein PHYSODRAFT_285961	Lipid metabolism
262108963	Glucosylceramidase	Lipid metabolism
262105919	3-ketodihydrosphingosine reductase	Lipid metabolism
262105742	Acyl-CoA dehydrogenase	Lipid metabolism
348688828	Hypothetical protein PHYSODRAFT_353568	Nucleobase-containing compound metabolism
348688657	hypothetical protein PHYSODRAFT_294028	Nucleobase-containing compound metabolis
348687452	Hypothetical protein PHYSODRAFT_284079	Nucleobase-containing compound metabolism
348684415	Hypothetical protein PHYSODRAFT_284882	Nucleobase-containing compound metabolism
348677381	Hypothetical protein PHYSODRAFT_354553	Nucleobase-containing compound metabolism
348677150	hypothetical protein PHYSODRAFT_503916	Nucleobase-containing compound metabolism
348673952	Hypothetical protein PHYSODRAFT_286691	Nucleobase-containing compound metabolism
348672301	hypothetical protein PHYSODRAFT_547952	Nucleobase-containing compound metabolism
348671618	Hypothetical protein PHYSODRAFT_435859	Nucleobase-containing compound metabolism
348670008	hypothetical protein PHYSODRAFT_347790	Nucleobase-containing compound metabolism
262107481	Pre-mRNA-splicing factor SF2	Nucleobase-containing compound metabolism
262106006	60S ribosomal protein L15-1	Nucleobase-containing compound metabolism
262104367	NADH-ubiquinone oxidoreductase, putative	Nucleobase-containing compound metabolism
262099101	Pre-mRNA-processing-splicing factor 8	Nucleobase-containing compound metabolism
262095673	hypothetical protein PITG_19772	Nucleobase-containing compound metabolism
348684155	hypothetical protein PHYSODRAFT_478148	Others
348684075	hypothetical protein PHYSODRAFT_349787	Others
348683892	hypothetical protein PHYSODRAFT_353864	Others
348683825	putative dehydratase	Others
348681277	hypothetical protein PHYSODRAFT_557322	Others
348673004	hypothetical protein PHYSODRAFT_354913	Others
348673003	hypothetical protein PHYSODRAFT_354912	Others
262105863	aldo/keto reductase family	Others
262103226	succinate semialdehyde dehydrogenase	Others
262099089	succinate dehydrogenase iron-sulfur protein	Others
262098735	alcohol dehydrogenase, putative	Others
348690141	hypothetical protein PHYSODRAFT_284543	Response to stimulus
348683864	Hypothetical protein PHYSODRAFT_353859	Response to stimulus
348672012	Elicitin	Response to stimulus
262110397	glutaredoxin [Phytophthora infestans T30-4]	Response to stimulus
262109962	Alkaline phosphatase	Response to stimulus
262106782	Superoxide dismutase 2	Response to stimulus
262101058	Metalloprotease family M17	Response to stimulus
262099848	Conserved hypothetical protein	Response to stimulus
348678388	ABC transporter ABCA1 lipid exporter family	Transport
348690807	Hypothetical protein PHYSODRAFT_349569	Unclassified
348690475	Hypothetical protein PHYSODRAFT_323696	Unclassified
348689826	Hypothetical protein PHYSODRAFT_252686	Unclassified
348688971	Hypothetical protein PHYSODRAFT_537442	Unclassified
348688710	Hypothetical protein PHYSODRAFT_477401	Unclassified
348688366	Hypothetical protein PHYSODRAFT_353487	Unclassified
348687330	Hypothetical protein PHYSODRAFT_284057	Unclassified
348683932	Hypothetical protein PHYSODRAFT_485543	Unclassified
348683032	Hypothetical protein PHYSODRAFT_253833	Unclassified
348681957	hypothetical protein PHYSODRAFT_329682	Unclassified
348679629	Putative aldehyde reductase	Unclassified
348677732	Hypothetical protein PHYSODRAFT_351217	Unclassified
348677176	Hypothetical protein PHYSODRAFT_544745	Unclassified
348676390	Hypothetical protein PHYSODRAFT_286458	Unclassified
348675944	Hypothetical protein PHYSODRAFT_286379	Unclassified
348675844	hypothetical protein PHYSODRAFT_561378	Unclassified
348675783	Hypothetical protein PHYSODRAFT_333832	Unclassified
348673781	Hypothetical protein PHYSODRAFT_354996	Unclassified
348671617	Putative endo-1,3-beta-glucanase	Unclassified
348670901	Hypothetical protein PHYSODRAFT_520792	Unclassified
348670499	Hypothetical protein PHYSODRAFT_564545	Unclassified
348670494	Hypothetical protein PHYSODRAFT_318600	Unclassified
348669879	Hypothetical protein PHYSODRAFT_258871	Unclassified
348669733	Pleiotropic drug resistance protein ABC superfamily	Unclassified
348667665	hypothetical protein PHYSODRAFT_340572	Unclassified
348664988	Hypothetical protein PHYSODRAFT_356224	Unclassified
262111960	Long-chain-fatty-acid-CoA ligase	Unclassified
262111199	Conserved hypothetical protein	Unclassified
262109829	Endoribonuclease L-PSP	Unclassified
262108642	Zinc finger CDGSH domain-containing protein 1	Unclassified
262107467	Conserved hypothetical protein	Unclassified
262107418	Elongation of very long chain fatty acids protein	Unclassified
262106687	Cytochrome P450	Unclassified
262106295	ketol-acid reductoisomerase	Unclassified
262105739	Cyclopropane-fatty-acyl-phospholipid synthase	Unclassified
262104643	NmrA-like family protein	Unclassified
262104423	conserved hypothetical protein	Unclassified
262102846	Conserved hypothetical protein	Unclassified
262102598	Conserved hypothetical protein	Unclassified
262102403	Electron transfer flavoprotein subunit alpha	Unclassified
262100267	mannitol dehydrogenase, putative	Unclassified
262100233	Conserved hypothetical protein	Unclassified
262099516	Estradiol 17-beta-dehydrogenase	Unclassified
262098991	Endoplasmic reticulum-Golgi intermediate compartment protein	Unclassified
262098869	conserved hypothetical protein	Unclassified
262098739	Electron transfer flavoprotein subunit beta	Unclassified
262097505	deoxyhypusine hydroxylase, putative	Unclassified
262096965	Conserved hypothetical protein	Unclassified
262096670	Conserved hypothetical protein	Unclassified
262096466	Conserved hypothetical protein	Unclassified
262096097	ATP-binding Cassette (ABC) Superfamily	Unclassified

## References

[b1] Leonian L. H. Stem and fruit blight of peppers caused by *Phytophthora capsici* sp. nov. Phytopathology 12, 401–408 (1922).

[b2] Kamoun S. *et al.* The Top 10 oomycete pathogens in molecular plant pathology. Mol. Plant Pathol. 16, 413–434 (2015). 25178392 10.1111/mpp.12190 PMC6638381

[b3] Satour M. & Butler E. A root and crown rot of tomato caused by *Phytophthora capsici* and *P. parasitica*. Phytopathology 57, 510–515 (1967).

[b4] Erwin D. C. & Ribeiro O. K. Phytophthora diseases worldwide. (American Phytopathological Society (APS Press), 1996).

[b5] Hausbeck M. K. & Lamour K. H. *Phytophthora capsici* on Vegetable Crops: Research Progress and Management Challenges. Plant Dis. 88, 1292–1303 (2004). 10.1094/PDIS.2004.88.12.1292 30795189

[b6] Tian D. & Babadoost M. Host range of *Phytophthora capsici* from pumpkin and pathogenicity of isolates. Plant Dis. 88, 485–489 (2004). 10.1094/PDIS.2004.88.5.485 30812651

[b7] Crossan D., Haasis F. & Ellis D. *Phytophthora* blight of summer squash. Plant Dis. Rep. 38, 557–559 (1954).

[b8] Café-Filho A., Duniway J. & Davis R. Effects of the frequency of furrow irrigation on root and fruit rots of squash caused by *Phytophthora capsici*. Plant Dis. 79, 44–48 (1995).

[b9] Café-Filho A. & Duniway J. Effect of location of drip irrigation emitters and position of *Phytophthora capsici* infections in roots on *Phytophthora* root rot of pepper. Phytopathology 86, 1364–1369 (1996).

[b10] Lamour K. H., Stam R., Jupe J. & Huitema E. The oomycete broad‐host‐range pathogen *Phytophthora capsici*. Mol. Plant Pathol. 13, 329–337 (2012). 22013895 10.1111/j.1364-3703.2011.00754.x PMC6638677

[b11] Ristaino J. B. & Johnston S. A. Ecologically based approaches to management of *Phytophthora* blight on bell pepper. Plant Dis. 83, 1080–1089 (1999). 10.1094/PDIS.1999.83.12.1080 30841127

[b12] Louws F., Lancaster M., Holmes G. & Driver J. Evaluation of fungicides and host resistance for control of *Phytophthora* crown rot of pepper, 1999. Fungic. Nematicide Tests 55, 188 (2000).

[b13] Zhu S. *et al.* Flumorph is a novel fungicide that disrupts microfilament organization in *Phytophthora melonis*. Phytopathology 97, 643–649 (2007). 18943584 10.1094/PHYTO-97-5-0643

[b14] Sun H., Wang H., Stammler G., Ma J. & Zhou M. Baseline sensitivity of populations of *Phytophthora capsici* from China to three carboxylic acid amide (CAA) fungicides and sequence analysis of cholinephosphotranferases from a CAA‐sensitive isolate and CAA‐resistant laboratory mutants. J. Phytopathol. 158, 244–252 (2010).

[b15] Ko W.-h. Hormonal heterothallism and homothallism in *Phytophthora*. Annu. Rev. Phytopathol. 26, 57–73 (1988).

[b16] Judelson H. S. Sexual reproduction in Oomycetes: biology, diversity and contributions to fitness In Oomycete genetics and genomics: diversity, interactions and research tools (eds Lamour K. & Kamoun S.) 121–138 (Hoboken, New Jersey: John Wiley & Sons Inc., 2009).

[b17] Lamour K. & Hausbeck M. Susceptibility of mefenoxam-treated cucurbits to isolates of *Phytophthora capsici* sensitive and insensitive to mefenoxam. Plant Dis. 87, 920–922 (2003). 10.1094/PDIS.2003.87.8.920 30812795

[b18] Dunn A. *et al.* Population structure and resistance to mefenoxam of *Phytophthora capsici* in New York State. Plant Dis. 94, 1461–1468 (2010). 10.1094/PDIS-03-10-0221 30743368

[b19] Meitz J. C., Linde C. C., Thompson A., Langenhoven S. & McLeod A. *Phytophthora capsici* on vegetable hosts in South Africa: distribution, host range and genetic diversity. Australas. Plant Path. 39, 431–439 (2010).

[b20] Gobena D., McGrath M. T. & Lamour K. Survival and spread of *Phytophthora capsici* on Long Island, New York. Mycol. Prog. 11, 761–768 (2012).

[b21] Bi Y. *et al.* Sexual reproduction increases the possibility that *Phytophthora capsici* will develop resistance to dimethomorph in China. Plant Pathol. 63, 1365–1373 (2014).

[b22] Meng Q. *et al.* Biological and genetic characterization of *Phytophthora capsici* mutants resistant to flumorph. Plant Pathol. 60, 957–966 (2011).

[b23] Santos P. M., Simões T. & Sá‐Correia I. Insights into yeast adaptive response to the agricultural fungicide mancozeb: a toxicoproteomics approach. Proteomics 9, 657–670 (2009). 19137554 10.1002/pmic.200800452

[b24] Hou Y., Zheng Z., Xu S., Chen C. & Zhou M. Proteomic analysis of *Fusarium graminearum* treated by the fungicide JS399-19. Pest. Biochem. Physiol. 107, 86–92 (2013). 10.1016/j.pestbp.2013.05.009 25149240

[b25] Pang Z. *et al.* Proteomic profile of the plant‐pathogenic oomycete *Phytophthora capsici* in response to the fungicide pyrimorph. Proteomics 15, 2972–2982 (2015). 25914214 10.1002/pmic.201400502

[b26] Blum M. *et al.* Mandipropamid targets the cellulose synthase‐like PiCesA3 to inhibit cell wall biosynthesis in the oomycete plant pathogen, Phytophthora infestans. Mol. Plant Pathol. 11, 227–243 (2010). 20447272 10.1111/j.1364-3703.2009.00604.x PMC6640402

[b27] Schomburg D. & Salzmann M. In Enzyme Handbook 4 (ed. Barman T. E.) 335–342 (Springer, 1991).

[b28] Martin K., McDougall B. M., McIlroy S., Chen J. & Seviour R. J. Biochemistry and molecular biology of exocellular fungal β-(1, 3)-and β-(1, 6)-glucanases. FEMS Microbiol. Rev. 31, 168–192 (2007). 17313520 10.1111/j.1574-6976.2006.00055.x

[b29] Mélida H., Sandoval-Sierra J. V., Diéguez-Uribeondo J. & Bulone V. Analyses of extracellular carbohydrates in oomycetes unveil the existence of three different cell wall types. Eukaryot. Cell 12, 194–203 (2013). 23204192 10.1128/EC.00288-12 PMC3571302

[b30] Tyler B. M. *et al.* *Phytophthora* genome sequences uncover evolutionary origins and mechanisms of pathogenesis. Science 313, 1261–1266 (2006). 16946064 10.1126/science.1128796

[b31] Yu B. & Howell P. L. Intragenic complementation and the structure and function of argininosuccinate lyase. Cell. Mol. Life Sci. 57, 1637–1651 (2000). 11092456 10.1007/PL00000646 PMC11147086

[b32] Whitfield C. D., Steers E. J. & Weissbach H. Purification and properties of 5-methyltetrahydropteroyltriglutamate-homocysteine transmethylase. J, Biol. Chem. 245, 390–401 (1970). 4904482

[b33] Murakoshi I., Kaneko M., Koide C. & Ikegami F. Enzymatic synthesis of the neuroexcitatory amino acid quisqualic acid by cysteine synthase. Phytochemistry 25, 2759–2763 (1986).

[b34] Jackson R. J., Hellen C. U. & Pestova T. V. The mechanism of eukaryotic translation initiation and principles of its regulation. Nat. Rev. Mol. Cell Bio. 11, 113–127 (2010). 20094052 10.1038/nrm2838 PMC4461372

[b35] Kim E. E. & Wyckoff H. W. Reaction mechanism of alkaline phosphatase based on crystal structures: two-metal ion catalysis. J. Mol. Biol. 218, 449–464 (1991). 2010919 10.1016/0022-2836(91)90724-k

[b36] El Hadrami A. *et al.* Plants versus fungi and oomycetes: pathogenesis, defense and counter-defense in the proteomics era. Int. J. Mol. Sci. 13, 7237–7259 (2012). 22837691 10.3390/ijms13067237 PMC3397523

[b37] Pawson T. Regulation and targets of receptor tyrosine kinases. Eur. J. Cancer 38, S3–S10 (2002). 12528767 10.1016/s0959-8049(02)80597-4

[b38] Kamoun S., Klucher K. M., Coffey M. D. & Tyler B. M. A gene encoding a host-specific elicitor protein of *Phytophthora parasitica*. Mol. Plant-Microbe Interact. 6, 573–573(1993). 8274771 10.1094/mpmi-6-573

[b39] Ricci P. *et al.* Structure and activity of proteins from pathogenic fungi *Phytophthora* eliciting necrosis and acquired resistance in tobacco. Eur. J. Biochem. 183, 555–563 (1989). 2776750 10.1111/j.1432-1033.1989.tb21084.x

[b40] Van’t Slot K. A. & Knogge W. A dual role for microbial pathogen-derived effector proteins in plant disease and resistance. Crit. Rev. Plant Sci. 21, 229–271 (2002).

[b41] Morris P. F. & Phuntumart V. Inventory and comparative evolution of the ABC superfamily in the genomes of *Phytophthora ramorum* and *Phytophthora sojae*. J. Mol. Evol. 68, 563–575 (2009). 19407922 10.1007/s00239-009-9231-8

[b42] Gan C. S., Chong P. K., Pham T. K. & Wright P. C. Technical, experimental, and biological variations in isobaric tags for relative and absolute quantitation (iTRAQ). J. Proteome Res. 6, 821–827 (2007). 17269738 10.1021/pr060474i

[b43] Xu D. *et al.* Serum protein S100A9, SOD3, and MMP9 as new diagnostic biomarkers for pulmonary tuberculosis by iTRAQ‐coupled two‐dimensional LC‐MS/MS. Proteomics 15, 58–67 (2015). 25332062 10.1002/pmic.201400366

[b44] Moulder R. *et al.* Quantitative proteomics analysis of the nuclear fraction of human CD4+ cells in the early phases of IL-4-induced Th2 differentiation. Mol. Cell. Proteomics 9, 1937–1953 (2010). 20467038 10.1074/mcp.M900483-MCP200 PMC2938108

[b45] Michalski A. *et al.* Mass spectrometry-based proteomics using Q Exactive, a high-performance benchtop quadrupole Orbitrap mass spectrometer. Mol. Cell. Proteomics 10, M111. 011015 (2011). 21642640 10.1074/mcp.M111.011015 PMC3284220

[b46] Colaert N. *et al.* Thermo-msf-parser: an open source Java library to parse and visualize Thermo Proteome Discoverer msf files. J. Proteome Res. 10, 3840–3843 (2011). 21714566 10.1021/pr2005154

[b47] Deutsch E. W. *et al.* A guided tour of the Trans‐Proteomic Pipeline. Proteomics 10, 1150–1159 (2010). 20101611 10.1002/pmic.200900375 PMC3017125

[b48] Delolme F. *et al.* Proteolytic control of TGF-β co-receptor activity by BMP-1/tolloid-like proteases revealed by quantitative iTRAQ proteomics. Cell. Mol. Life Sci. 72, 1009–1027 (2015). 25260970 10.1007/s00018-014-1733-x PMC11113849

[b49] Ye J. *et al.* WEGO: a web tool for plotting GO annotations. Nucleic Acids Res. 34, W293–W297 (2006). 16845012 10.1093/nar/gkl031 PMC1538768

[b50] Liu J., Chen L., Wang J., Qiao J. & Zhang W. Proteomic analysis reveals resistance mechanism against biofuel hexane in *Synechocystis* sp. PCC 6803. Biotechnol. Biofuels 5, 68 (2012). 22958739 10.1186/1754-6834-5-68 PMC3479031

