# Supplementary material for: Insights into the adaptive response of the plant-pathogenic oomycete Phytophthora capsici to the fungicide flumorph
Source: Sci Rep. 2016 Apr 6;6:24103. doi: 10.1038/srep24103 (PMC4822174; doi:10.1038/srep24103)
Supplement: Supplementary Information [file srep24103-s1.pdf]

Insights into the adaptive response of the plant-pathogenic oomycete *Phytophthora capsici* to the fungicide flumorph

Zhili Pang, Lei Chen, and Xili Liu

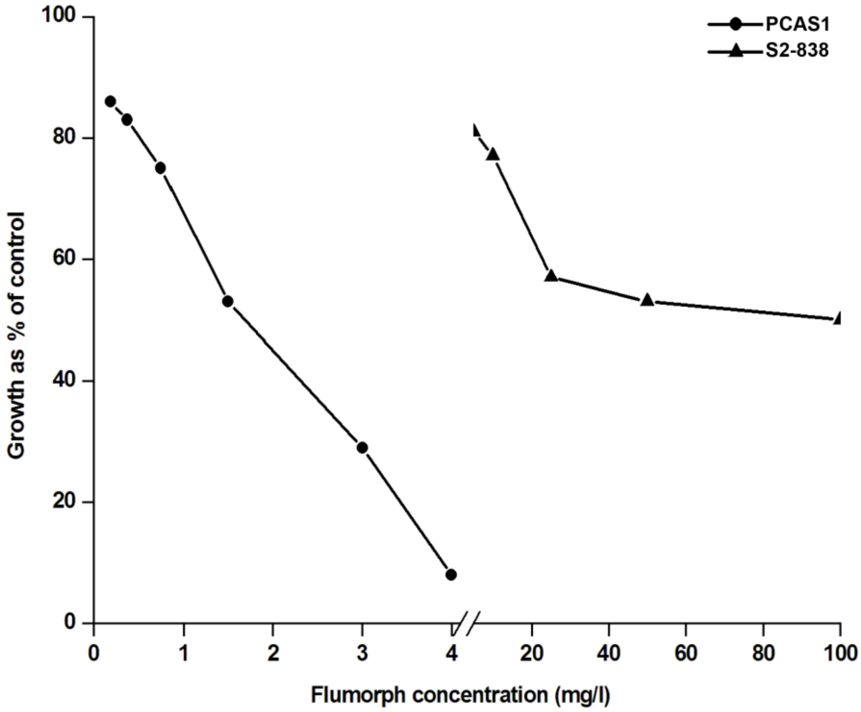

Supplementary Fig. S1 Sensitivity of hyphal growth of wild-type isolate PCAS1 and flumorph-resistant isolate S_2-838 of *Phytophthora capsici* to flumorph.
